# Importance of Decision Support Implementation in Emergency Department Vancomycin Dosing

**DOI:** 10.5811/westjem.2015.4.25760

**Published:** 2015-06-22

**Authors:** Brett Faine, Nicholas Mohr, Kari K. Harland, Kathryn Rolfes, Blake Porter, Brian M. Fuller

**Affiliations:** * University of Iowa Hospitals and Clinics, Department of Emergency Medicine, Iowa City, Iowa; † University of Iowa College of Pharmacy, Iowa City, Iowa; ‡ Washington University, Department of Anesthesiology, St. Louis, Missouri

## Abstract

**Introduction:**

The emergency department (ED) plays a critical role in the management of life-threatening infection. Prior data suggest that ED vancomycin dosing is frequently inappropriate. The objective is to assess the impact of an electronic medical record (EMR) intervention designed to improve vancomycin dosing accuracy, on vancomycin dosing and clinical outcomes in critically ill ED patients.

**Methods:**

Retrospective before-after cohort study of all patients (n=278) treated with vancomycin in a 60,000-visit Midwestern academic ED (March 2008 and April 2011) and admitted to an intensive care unit. The primary outcome was the proportion of vancomycin doses defined as “appropriate” based on recorded actual body weight. We also evaluated secondary outcomes of mortality and length of stay.

**Results:**

The EMR dose calculation tool was associated with an increase in mean vancomycin dose ([14.1±5.0] vs. [16.5±5.7] mg/kg, p<0.001) and a 10.3% absolute improvement in first-dose appropriateness (34.3% vs. 24.0%, p=0.07). After controlling for age, gender, methicillin-resistant *staphylococcus aureus* infection, and Acute Physiology and Chronic Health Evaluation II score, 28-day in-hospital mortality (odds ratio OR 1.72; 95% CI [0.76–3.88], p=0.12) was not affected.

**Conclusion:**

A computerized decision-support tool is associated with an increase in mean vancomycin dose in critically ill ED patients, but not with a statistically significant increase in therapeutic vancomycin doses. The impact of decision-support tools should be further explored to optimize compliance with accepted antibiotic guidelines and to potentially affect clinical outcome.

## INTRODUCTION

Vancomycin is a glycopeptide antibiotic that exhibits time-dependent killing. It has been used for more than five decades to treat resistant organisms, such as methicillin-resistant *Staphylococcus aureus* (MRSA), and in the empiric treatment for severe sepsis and septic shock. Efficacy is often predicted by the ratio of the area under the antibiotic concentration curve and the minimum inhibitory concentration of the infecting pathogen (AUC/MIC ratio). An AUC/MIC ratio of ≥400 with trough serum concentrations of 15–20mg/L are recommended to achieve clinical effectiveness and limit the development of resistant microorganisms. 1 MRSA vancomycin treatment failures are occurring with increasing frequency, and vancomycin-intermediate *Staphylococcus aureus* (VISA) has emerged as a leading cause of vancomycin failures and poor clinical outcomes. 2 – 3

Inappropriate vancomycin dosing is associated with the emergence of VISA. 4–6 Conventional dosing practices initiate vancomycin at 1000mg every 12 hours. 7 Due to the association of conventional dosing and subtherapeutic vancomycin trough levels, however, current guidelines advocate for weight-based dosing algorithms. 1, 8

The emergency department (ED) plays a critical role in the management of life-threatening infection. 9 There is also an increased awareness of the ED’s role in antimicrobial initiation, with an increased interest in antibiotic stewardship beginning in the ED. 10 ED antibiotic initiatives include both appropriate usage and timely administration. Prior data suggest that ED dosing of vancomycin is frequently inappropriate, yet vancomycin administered in the ED is often continued into the inpatient course. 7, 11 This suggests that the ED is highly influential on overall antibiotic therapy, regardless of dosing or indication appropriateness. 7 This practice pattern has the potential for developing antibiotic resistance, as organisms such as VISA are invariably associated with vancomycin exposure and subtherapeutic dosing strategies. 4

Appropriate antibiotic selection and dose optimization is a prime determinant of outcome in critically ill patients. 12 ED clinical pharmacists improve appropriate antibiotic dosing, yet fewer than 5% of EDs have an ED-based pharmacist. 10, 13 Therefore, an electronic medical record (EMR) based antibiotic stewardship strategy could be a generalizable intervention with a measurable effect on antibiotic selection and dosing across many EDs in the community.

The primary objective of this analysis was to assess the impact of an EMR intervention on vancomycin dosing accuracy in critically ill ED patients. We hypothesized that an EMR intervention would be associated with improvement in vancomycin dosing accuracy. Secondary objectives were to assess the impact of vancomycin dosing on mortality, hospital length of stay, acute kidney injury, and the impact of obesity on vancomycin dosing accuracy.

## METHODS

### Patients and Setting

This study was a retrospective before-after cohort study (March 2008–May 2009 [before] and November 2009–April 2011 [after]) conducted in the ED of a Midwestern academic Level I trauma center with an annual ED census of 60,000 patient visits.

### Intervention

**Figure 1:**
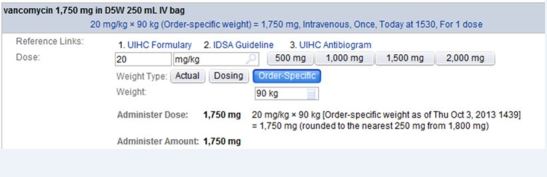
Revised order in EMR. Providers see the recommended dose based on the computer calculation. The computer recommended first dose is 20mg/kg actual body weight with maximum dose 2 grams. *EMR,* electronic medical record; *IDSA*, Infectious Diseases Society of America; *UHC*, University Health System Consortium

We included all patients treated with vancomycin in the ED and admitted to an intensive care unit. For patients who received vancomycin on multiple ED visits during the study period (2%), only the first visit was included in the analysis. The post-intervention period began after weight-based vancomycin dosing guidance was incorporated into computerized physician order entry (CPOE) to correspond to updated guidelines from the Infectious Diseases Society of America in 2009. 1, 14 The EMR intervention included an automatic dose calculation tool included in the CPOE order, and an educational campaign (e-mail notification to all EM staff and a presentation by a research team member to EM residents and attending physicians) accompanied the rollout. The automatic dose calculation tool recommended a vancomycin dose of 20mg/kg actual body weight (as recorded in the medical record). The calculated dose was rounded to the nearest 250mg and did not recommend greater than 2 gram in a single dose (Figure 1). A six-month run-in period was excluded from analysis *a priori* to assure that all providers had time to acclimate themselves to the new automatic dose calculation tool.

### Data Abstraction

We abstracted vancomycin dosing and clinical variables from the EMR using both database query and manual data collection by two trained data abstractors (KD, BP). The two data abstractors were blinded to the study hypothesis and received formal training in proper data abstraction techniques. After data abstraction, 15% of charts were randomly selected for review by a third independent investigator (BAF) to validate data accuracy and abstraction techniques. We defined all variables *a priori* and recorded them in an electronic database for analysis.

### Definitions

Appropriate vancomycin dose was defined as 15–20mg/kg in accordance with guideline recommendations. 1 We based obesity categorization on the definitions by the World Health Organization as underweight (body mass index (BMI) <18.5), normal (18.5–24.99), overweight (25.0–29.99) and obese (≥30). 15 Mortality was assessed at 28 days after hospital admission. Subjects discharged alive before 28 days were coded as alive. We defined acute kidney injury as increase in serum creatinine by 0.3mg/dL within 48 hours or increase to 1.5 times baseline. 16 We calculated Acute Physiology and Chronic Health Evaluation II (APACHE-II) scores based on clinical data collected within 24 hours of hospital admission. Parameters not recorded were imputed to be normal for the purposes of APACHE-II calculation. Vancomycin levels were collected during each patient’s hospital stay.

### Outcomes

The primary outcome was the proportion of vancomycin doses defined as “appropriate” based on recorded actual body weight. Secondary outcomes included 28-day in-hospital mortality, hospital length of stay and acute kidney injury (safety outcome). We also measured the impact of obesity and the sustained effect of the intervention (stratified in four-month intervals). Overweight and obese patients who received the maximum dose of 2 gram were categorized in the “appropriate” group even though the calculator recommended larger doses based on the actual weight.

We conducted univariate analysis using t-test, chi-squared test, or ANOVA, as appropriate. Multivariable logistic regression analysis was used to estimate the effect of the EMR intervention on 28-day in-hospital mortality, controlling for potentially confounding covariates (age, sex, MRSA, BMI, APACHE II score, acute kidney injury, vasopressor administration, mechanical ventilation and history of hemodialysis). We prespecified variables included in the model based on *a priori* knowledge and defined a statistical threshold of p<0.20. Collinearity and statistical interactions were measured. All tests were two-tailed and a p-value <0.05 was considered statistically significant. We conducted all analyses using SAS® software (version 9.3, SAS System for Microsoft, SAS Institute Inc., Cary, NC, USA). The institutional review board approved the study protocol.

## RESULTS

**Figure 2:**
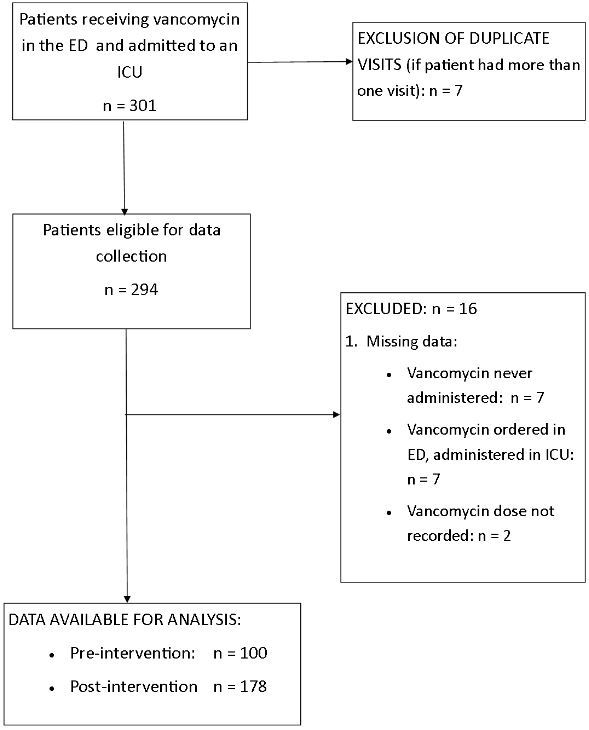
Pre- and post-intervention vancomycin administration and eligible patients for analysis flow diagram. *ICU,* intensive care unit

We included 278 subjects in the study (Figure 2). 17 Baseline characteristics are shown in Table 1. Mean vancomycin dose increased after the intervention ([14.1±5.0] vs. [16.5±5.7]mg/kg, p<0.001). First-dose appropriateness increased from 24.0% to 34.3%, p=0.07. Overall, 30.6% of patients received an appropriate dose (Table 2). The proportion of patients receiving a dose of 1g decreased (84% vs. 52%, p<0.001). Vancomycin trough levels were obtained in 157 patients (56%), and median trough levels did not change during the study period (13.3, IQR [10.6–22.4]) vs. 13.8, IQR [9.4–18.3], p=0.59)

Twenty-eight day mortality (10.0% vs. 16.9%, p=0.12) did not change with the intervention. In univariate analysis, mortality was not associated with the intervention period (Table 3). Using multivariable logistic regression to adjust for age, sex, MRSA infection status, and APACHE-II, 28-day mortality was not associated with the EMR intervention (adjusted odds ratio 1.72 [0.76–3.88], p=0.12). The intervention did not significantly increase the risk of acute kidney injury in the post intervention group (34.0% vs. 31.5%, p=0.66). The appropriateness of the vancomycin dose did not have a significant effect on hospital length of stay for the pre- and post-intervention groups (Table 2).

**Figure 3:**
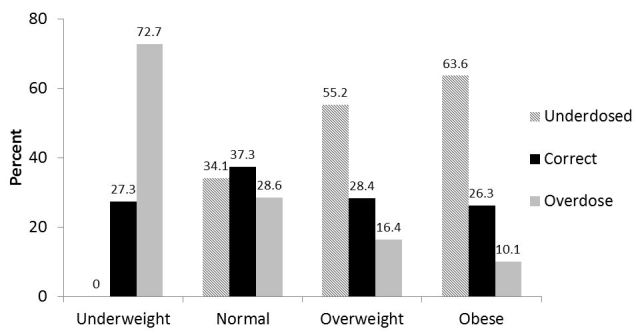
Appropriateness of vancomycin dose by patient weight.

Obesity had a significant effect on the appropriateness of vancomycin dosing (Figure 3). Overweight (55.2% vs. 34.1%) and obese (63.6% vs. 34.1%) subjects were more likely to be underdosed (p<0.0001), and no underweight patients were underdosed. Underweight patients were more like to receive an inappropriately high dose than the normal weight patients (72.7% vs. 28.6%, p<0.0001).

The prevalence of MRSA identified as an infectious agent from a blood culture or bronchoalveolar lavage increased between study periods from 5.0% to 13.5% (p=0.03). Among subjects without MRSA, neither inappropriately low nor high doses were associated with survival (Table 3).

## DISCUSSION

As a recommended therapy for critically ill patients with life-threatening infection, vancomycin is frequently administered in the ED. Although other investigators have examined the role of vancomycin dosing on clinical outcomes, our study evaluated systematically the effect of an EMR intervention on the clinical outcome of patients admitted from the ED to an intensive care unit. This is an important finding because it highlights both the role of quality improvement initiatives and their unintended consequences on clinical outcomes.

In our cohort, the EMR intervention increased the dose of vancomycin (14.1±5.0mg/kg vs. 16.5±5.7mg/kg, p<0.0001). The increase in the mean vancomycin dose was relatively small; however, the proportion of patients who received a dose recommended by Infectious Diseases Society of America (IDSA) guidelines increased in the post-intervention group. Sixty-six percent of patients received a dose recommended by the algorithm but outside the IDSA-recommended vancomycin range because the rounding pushed doses inappropriately high for some patients. Even though we decreased “traditional” (1 gram) dosing, a button on the vancomycin order still permitted easy prescribing of this dose, so the rate of traditional dosing still remained over 50%.

The only clinical predictor that had a significant effect on vancomycin dosing in the post-intervention group was patient weight. Overweight and obese patients were more likely to be underdosed. This occurred even with our analysis categorizing overweight and obese patients as receiving the “appropriate” dose if they received the maximum 2 gram dose even though the calculator recommended a higher dose based on their actual body weight. Another factor contributing to underdosing may be the fear of nephrotoxicity. A main objective of vancomycin dosing is to achieve therapeutic trough levels rapidly. A recent meta-analysis reported the incidence of nephrotoxicity between 5% and 43%, and suggested that the rate is low without concomitant administration of other nephrotoxins. 18 In our patient population, increased patient weights led to unreliable dosing.

The EMR intervention increased the dose of vancomycin but failed to have significant effects on clinical outcomes. The increased dose in the post-intervention group did not have a significant effect on mortality, hospital length of stay or increase the risk of acute kidney injury. There was a trend towards increased mortality in the post-intervention group (as reported in a prior study) but this did not reach statistical significance. 7

One of the most speculative aspects of our study is the association of mortality with a change in drug dosing. A prior study suggested that higher vancomycin dosing was associated with higher mortality. 7 Two interpretations of this observation are possible: either sicker patients were treated with higher doses (bias), or vancomycin actually impairs survival among patients without vancomycin-treated infection. Interestingly, most reports of increased effectiveness of aggressive vancomycin dosing enroll only patients with documented vancomycin-susceptible infection (e.g., MRSA). 8, 20 If vancomycin improves survival among MRSA patients but harms patients without MRSA, the population prevalence of MRSA would be the primary determinant of effectiveness in a study. Furthermore, such a model would suggest that vancomycin only benefits population survival if the local incidence of MRSA exceeds a threshold. Although our study does not confirm the prior finding, it was not powered to detect a difference in mortality. 7 The nonsignificant effect estimate, however, closely mirrors the effect size of increased mortality with higher vancomycin dosing in the previous study. The before-after methodology of this analysis better limits the potential for bias. Based on these data, it is imperative that real time diagnostics are developed to avoid exposure to unnecessary therapies in critically ill patients.

Instituting an EMR intervention can significantly decrease dosing errors and improve compliance with recommended dosing. 21 However, EMR interventions can also have unintended consequences, including dosing errors. An unintended effect of our intervention was that it increased the proportion of patients receiving a dose higher than recommended. Fortunately, the higher doses did not result in an increase in adverse events. In our study, administering modestly higher doses did not increase acute kidney injury, but inpatient dosing regimens were not characterized.

## LIMITATIONS

Our study has several important limitations. First, this was a retrospective data analysis, which introduces a risk of bias due to poor documentation or incomplete information. We selected variables that would have been available at the time of the ED visit and were likely to be documented accurately in the EMR. Even with these measures, some relevant factors may not have been captured. Second, our study was carried out at a single center with a relatively low risk of MRSA. The prevalence of MRSA in our study did increase between study periods, which is consistent with other reports in the United States. 22 Since the primary outcome was provider behavior, our findings are likely valid. 6 Third, we used a run-in design which excluded a six-month time frame used for education for the EMR intervention and a systematic shift to weight-based dosing. By excluding this time frame we could have underestimated early adverse effects of the clinical change. Fourth, we were unable to gauge appropriateness of the indication for vancomycin in our ED. One study evaluated the appropriateness of vancomycin in the ED and found that 40% of the patients in the study did not warrant vancomycin administration. 6 Last, based on the recommendations from the IDSA clinical practice guideline for the treatment of MRSA, we elected to cap the dose of vancomycin at 2 grams for all patients in the intervention group. 14 Vancomycin pharmacokinetics (volume of distribution, protein binding, and clearance) can be altered in obese patients; however, the variability does not mean that obese patients require higher total daily doses to attain target trough concentrations. 23 – 24

## CONCLUSION

In conclusion, the prevalence of therapeutic vancomycin dosing (goal 15–20mg/kg actual body weight) did not change after the implementation of a decision support system and an automated EMR dose calculator. Higher vancomycin dosing post-intervention was not associated with acute kidney injury or 28-day in-hospital mortality. Additional specific decision-support interventions (including removing the option to select non-recommended dosing) should be explored to further increase compliance with accepted guidelines to improve antibiotic dosing practices in the ED.

## Figures and Tables

**Table 1:** Patient demographics, outcomes and vancomycin dosing before and after an electronic medical record intervention.

	EMR intervention			
	
	Total	Before	After	p-value
Total, n(%)	278	100 (36.0)	178 (64.0)	
Age, y (SD)	57.2 (17.7)	57.5 (17.5)	57.1 (17.8)	0.85
Male, n(%)	172 (61.9)	58 (58.0)	114 (64.0)	0.32
BMI, kg/m^2^ (SD)	30.1 (13.1)	31.9 (18.8)	29.1 (9.0)	0.18
APACHE II, score (SD)	17.7 (5.3)	17.8 (5.4)	17.6 (5.2)	0.84
Vancomycin dosing				
Total, mean (SD)	1253.6 (381.2)	1115 (283)	1331.5 (407)	<0.0001
mg/kg, mean (SD)	15.7 (5.6)	14.1 (5.0)	16.5 (5.7)	0.0003
Patients given 1 gram, n(%)	166 (59.7)	84 (84.0)	92 (51.7)	<0.0001
Appropriate dose, n(%)	85 (30.6)	24 (24.0)	61 (34.3)	0.0745
MRSA in culture, n(%)	29 (10.4)	5 (5.0)	24	0.03
Acute kidney injury, n(%)	90 (32.4)	34 (34.0)	56	0.66
28 day in-hospital mortality, n(%)	40	10 (10.0)	30	0.12

*EMR*, electronic medical record; *APACHE*, acute physiology and chronic health evaluation; *MRSA*, methicillin-resistant *Staphylococcus aureus; BMI*, body mass index

**Table 2:** Patient demographics and outcomes by appropriateness of vancomycin dose (n=278).

	Appropriateness of vancomycin dose			
	
	Underdosed (n=138)	Correct (n=85)	Overdosed (n=55)	p-value
Age, y (SD)	60.9 (16.1)	54.8 (16.8)	51.8 (20.7)	0.0015
Male, n(%)	93 (67.4)	41 (48.2)	38 (69.1)	0.0078
BMI, kg/m^2^ (SD)	32.9 (12.9)	28.3 (9.2)	25.9 (16.8)	0.0015
APACHE II, score (SD)	18.2 (5.1)	17.1 (5.2)	17.1 (5.7)	0.22
Acute kidney injury, n(%)	47 (34.0)	28 (32.9)	15 (27.3)	0.66
Post-EMR intervention, n(%)	72 (52.2)	61 (71.8)	45 (81.8)	0.0001
Length of stay, days (SD)	11.5 (13.7)	11.2 (11.5)	9.5 (9.3)	0.56
28 day in-hospital mortality, n(%)	18 (13.0)	13 (15.3)	9 (16.4)	0.81

*APACHE,* acute physiology and chronic health evaluation; *EMR,* electronic medical record; *BMI*, body mass index

**Table 3:** Unadjusted and adjusted odds of 28-day in-hospital mortality among patients receiving vancomycin (n=278) and adjusted odds of 28-day in-hospital mortality among those without methicillin-resistant *Staphylococcus aureus* (MRSA).

	28-day in-hospital mortality				
				
	No^1^ n (%)	Yes^2^ n (%)	p-value^3^	OR (95% CI)	Adjusted OR (95% CI)^5^
Age, y [SD]	56.0 [17.5]	64.7 [17.1]	0.0040	1.03 (1.01–1.05)	1.03 (1.01–1.06)
Sex					
Female	96 (40.3)	10 (25.0)	0.06	1.0 (ref)	1.0 (ref)
Male	142 (59.7)	30 (75.0)		2.03 (0.95–4.34)	2.29 (1.02–5.14)
MRSA					
No	213 (89.5)	36 (90.0)	0.92	1.0 (ref)	1.0 (ref)
Yes	25 (10.5)	4 (10.0)		0.95 (0.31–2.89)	0.76 (0.24–2.41)
BMI, kg/m^2^ [SD]	30.4 [11.8]	28.1 [19.4]	0.47	0.98 (0.94–1.02)	
SBP, mmHg [SD]	115.7 [29.3]	113.9 [34.6]	0.73	1.0 (0.99–1.01)	
APACHE II, score [SD]	17.4 [5.4]	19.2 [4.4]	0.05	1.07 (1.00–1.14)	1.04 (0.96–1.11)
Acute kidney injury					
No	165 (69.3)	23 (57.5)	0.14	1.0 (ref)	
Yes	73 (30.7)	17 (42.5)		1.67 (0.84–3.31)	
Vasopressors					
No	213 (89.5)	33 (82.5)	0.20	1.0 (ref)	
Yes	25 (10.5)	7 (17.5)		1.81 (0.72–4.51)	
Intubation					
No	201 (84.5)	29 (72.5)	0.06	1.0 (ref)	
Yes	37 (15.5)	11 (27.5)		2.06 (0.95–4.48)	
History of dialysis					
No	221 (92.9)	39 (97.5)	0.49^4^	Unable to calc	
Yes	17 (7.1)	1 (2.5)			
Post-EMR intervention					
No	90 (37.8)	10 (25.0)	0.12	1.0 (ref)	1.0 (ref)
Yes	148 (62.2)	30 (75.0)		1.82 (0.85–3.91)	1.72 (0.76–3.88)
Appropriate vancomycin dose					
Underdosed	120 (50.4)	18 (45.0)	0.81	0.83 (0.38–1.80)	0.60 (0.26–1.41)
Correct	72 (30.3)	13 (32.5)		1.0 (ref)	1.0 (ref)
Overdosed	46 (19.3)	9 (22.5)		1.08 (0.43–2.74)	0.88 (0.33–2.37)
Vancomycin dosing					
Total, mean [SD]	1260.5 [384.8]	1212.5 [360.5]	0.46	1.0 (0.999–1.001)	
Mg/kg, mean [SD]	15.5 [5.6]	16.6 [5.2]	0.24	1.04 (0.98–1.10)	

*APACHE,* acute physiology and chronic health evaluation; *EMR,* electronic medical record; *BMI*, body mass index

Brackets denotes standard deviation. Parenthesis denotes percentage.

1 n=238.

2 n=40.

3 Chi-square test for categorical variables and student’s t-test for continuous variables.

4 Fisher’s exact test.

5 Model is adjusted for all variables that have an adjusted odds ratio reported.
